# Discrepant post filter ionized calcium concentrations by common blood gas analyzers in CRRT using regional citrate anticoagulation

**DOI:** 10.1186/s13054-015-1027-1

**Published:** 2015-09-08

**Authors:** Patrik Schwarzer, Sven-Olaf Kuhn, Sylvia Stracke, Matthias Gründling, Stephan Knigge, Sixten Selleng, Maximilian Helm, Sigrun Friesecke, Peter Abel, Anders Kallner, Matthias Nauck, Astrid Petersmann

**Affiliations:** Institute of Clinical Chemistry and Laboratory Medicine, University Medicine Greifswald, Ferdinand-Sauerbruch-Strasse, 17475 Greifswald, Germany; Department of Anaesthesiology and Intensive Care Medicine, University Medicine Greifswald, Greifswald, Germany; Department of Internal Medicine A, University Medicine Greifswald, Greifswald, Germany; Department of Internal Medicine B, University Medicine Greifswald, Greifswald, Germany; Karolinska University Hospital, Stockholm, Sweden

## Abstract

**Introduction:**

Ionized calcium (iCa) concentration is often used in critical care and measured using blood gas analyzers at the point of care. Controlling and adjusting regional citrate anticoagulation (RCA) for continuous renal replacement therapy (CRRT) involves measuring the iCa concentration in two samples: systemic with physiological iCa concentrations and post filter samples with very low iCa concentrations. However, modern blood gas analyzers are optimized for physiological iCa concentrations which might make them less suitable for measuring low iCa in blood with a high concentration of citrate. We present results of iCa measurements from six different blood gas analyzers and the impact on clinical decisions based on the recommendations of the dialysis’ device manufacturer.

**Method:**

The iCa concentrations of systemic and post filter samples were measured using six distinct, frequently used blood gas analyzers. We obtained iCa results of 74 systemic and 84 post filter samples from patients undergoing RCA for CRRT at the University Medicine of Greifswald.

**Results:**

The systemic samples showed concordant results on all analyzers with median iCa concentrations ranging from 1.07 to 1.16 mmol/L. The medians of iCa concentrations for post filter samples ranged from 0.21 to 0.50 mmol/L. Results of >70 % of the post filter samples would lead to major differences in decisions regarding citrate flow depending on the instrument used.

**Conclusion:**

Measurements of iCa in post filter samples may give misleading information in monitoring the RCA. Recommendations of the dialysis manufacturer need to be revised. Meanwhile, little weight should be given to post filter iCa. Reference methods for low iCa in whole blood containing citrate should be established.

## Introduction

**Fig. 1:**
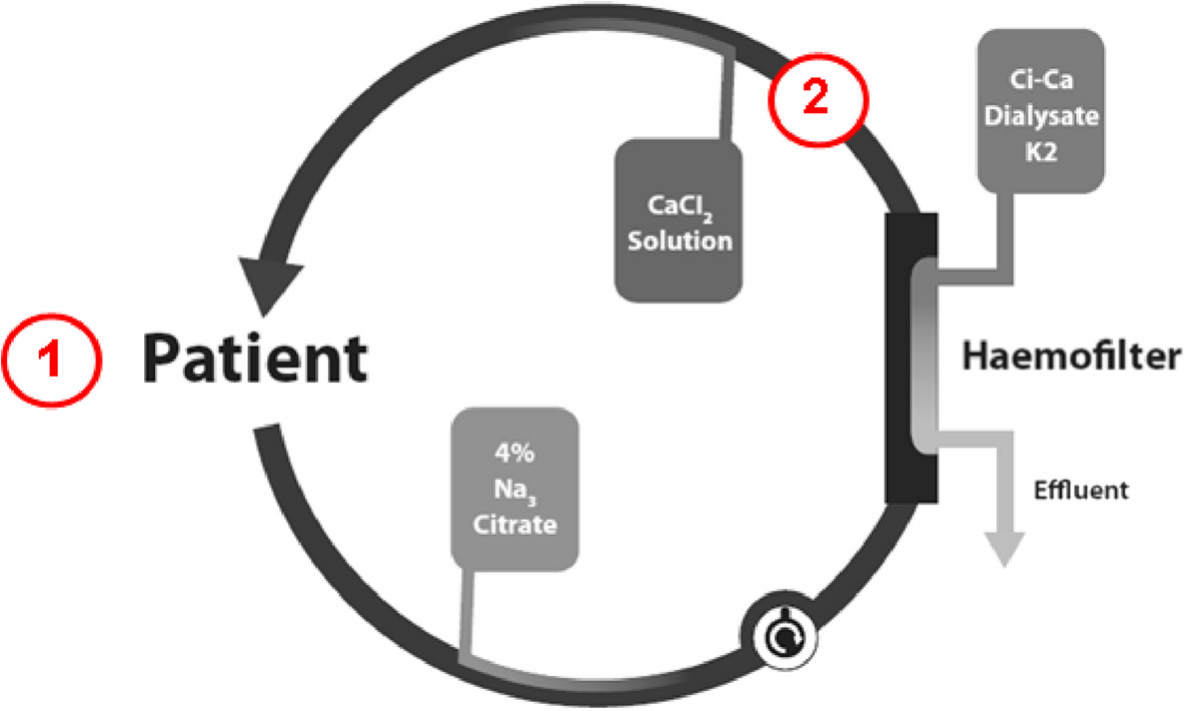
Sampling points for ionized calcium (iCa) concentration measurements in regional citrate anticoagulation for continuous renal replacement therapy. Blood samples for iCa concentration measurements in systemic samples are taken in 1) and blood samples for iCa concentrations measurements in post filter samples are taken in 2)

Regional citrate anticoagulation is recommended by the Kidney Disease Improving Global Outcomes Clinical Practice Guidelines for continuous renal replacement therapy (CRRT) in critically ill patients [1]. Regional citrate anticoagulation (RCA) for CRRT represents a well-accepted alternative of anticoagulation for patients with enhanced bleeding risk due to systemic anticoagulation [2–4] by limiting the anticoagulation with citrate to the extra corporeal circulation. Anticoagulation in CRRT is achieved by administering citrate immediately after the blood enters the extracorporeal circulation. The anticoagulant effect results from chelating ionized calcium (iCa) with citrate in the extra corporeal circulation leading to reduced iCa concentrations. The chelated calcium cannot act as a cofactor in the coagulation cascade. The coagulation is distorted if iCa is below 0.5 mmol/L and completely inhibited if the concentration drops below 0.3 mmol/L [5, 6]. When an adequate amount of citrate is added (citrate flow) dialysis can take place through a filter without clotting. After filtration calcium is added (calcium flow) to the dialyzed blood in order to raise iCa concentrations back to a physiological level and reconstitute coagulation. ICa concentration measurements (Fig. 1) are recommended by the dialysis manufacturer in samples collected after the dialysis filter (post filter samples) and directly from the patient (systemic samples) [7, 8].

The clinical effectiveness depends on fine-tuning of the systemic iCa concentration, which is modified by the calcium flow, and the post filter iCa concentration, which is affected by the citrate flow. It is recommended by the manufacturer (Fresenius Medical Care, Bad Homburg, Germany) to analyze the systemic as well as the post filter iCa concentrations [7, 8]. Based on these iCa measurements the manufacturer advises schemes for adjusting both citrate and calcium flows. No recommendations are given by the manufacturer on methods and instruments for iCa measurements. Many iCa measurements in the core laboratory are performed using blood gas analyzers which are often also used in point of care testing (POCT). However, modern blood gas analyzers are optimized for physiological iCa concentrations, e.g. blood without additions of excessive electrolytes or extreme concentrations of such components. The matrix of the post filter sample is characterized by un-physiologically high citrate and extremely low iCa concentrations. This might make currently available instruments less suitable for these post filter samples and thus not fit for purpose as required by regulatory bodies and Good Laboratory Practice. This applies to current instruments whether used in core laboratories or POCT.

The present study was triggered by a simultaneous change of instruments in both the core laboratory and on the wards. The laboratory, which is responsible for the POCT devices, and the blood gas instrument manufacturer were unaware of the recommended measurement of post filter samples. Accordingly, the blood gas instruments were not validated for these measurements and the CE approval did not cover this application. Method comparisons between the old and new devices showed remarkable differences in the post-filter samples.

The systemic iCa concentration interval is targeted to physiological concentrations (1.12–1.20 mmol/L) whereas the post filter iCa concentration is extremely low with a recommended target interval of 0.25–0.34 mmol/L [7, 8]. Reference methods for measuring iCa in whole blood samples for physiological iCa concentrations have been described [9]. Reference methods for low iCa and high citrate concentrations, as in post filter samples, are lacking so far. POCT and core laboratory iCa methods are not designed nor thoroughly evaluated for these conditions. We compared iCa concentration measurements of systemic and post filter samples from patients undergoing RCA for CRRT using six different commercially available blood gas analyzers and describe the potential impact of these analytical results on clinical decisions.

## Materials and methods

Six distinct blood gas instruments (GEM Premier 4000, Instrumentation Laboratory, Bedford, USA; pHOx and Prime, Nova Biomedical, Rödemark, Germany; ABL90, Radiometer, Copenhagen, Denmark; Cobas b 123, Roche Diagnostics, Mannheim, Germany; RAPID Point 500, Siemens Healthcare Diagnostics, Eschborn, Germany) were used to measure iCa concentrations in leftover material from samples obtained from patients undergoing RCA for CRRT (mulitFiltrate Ci-Ca® Continuous Veno-Venous Hemo Dialysis, CVVHD, Fresenius Medical Care, Bad Homburg, Germany) in the University Medicine of Greifswald from June to July 2014. The measurement intervals for iCa were 0.10–5.00 mmol/L (GEM4000), 0.10–2.70 mmol/L (Prime), 0.10–2.70 mmol/L (pHOx), 0.20–9.99 mmol/L (ABL90), 0.10–2.50 mmol/L (Cobas b 123), 0.20–5.00 mmol/L (RAPID Point 500). Measurements for monitoring RRT were performed using the ABL90 (Radiometer, Copenhagen, Denmark).

Systemic and post filter samples were used directly after measurements for clinical purposes had been completed, if the amount of the material left was sufficient. Sample material left was immediately brought to a nearby separate room with the instruments evaluated in the study. Therefore the pre-analytical phases were the same for all study samples. The measurements on all study instruments were completed within 10 minutes. The samples were not divided because the same syringe was used to feed the sample into each analyzer. The measurements were thus carried out more or less in parallel. Results of this study were not made available for patient care. The order of measurements was rotated between the blood gas analyzers according to a previously randomized scheme.

Ethical approval by the local ethical board (University of Greifswald; III UV 39/03) was obtained to use spare material from anonymized routine patient samples for evaluation and comparison purposes. For the use of anonymized spare material no informed consent from the patients was necessary. Statistical evaluations were performed using Microsoft Excel (2010).

## Results

A total of 218 samples (102 systemic and 116 post filter samples) from 29 patients were included in the study. Of these there were 74 systemic and 84 post filter samples with results for at least five of the devices. All internal quality controls (IQC) for iCa in the six blood gas analyzers complied with national regulations (Guideline of the German Medical Association on Quality Assurance in Medical Laboratory Examinations; Rili-BAEK) with a root mean square (a combined measure of imprecision and bias) below 7.5 % (IQC target values >1–2.5 mmol/L) and 14 % (IQC targets values from 0.2 to 1 mmol/L), respectively [10].

**Fig. 2:**
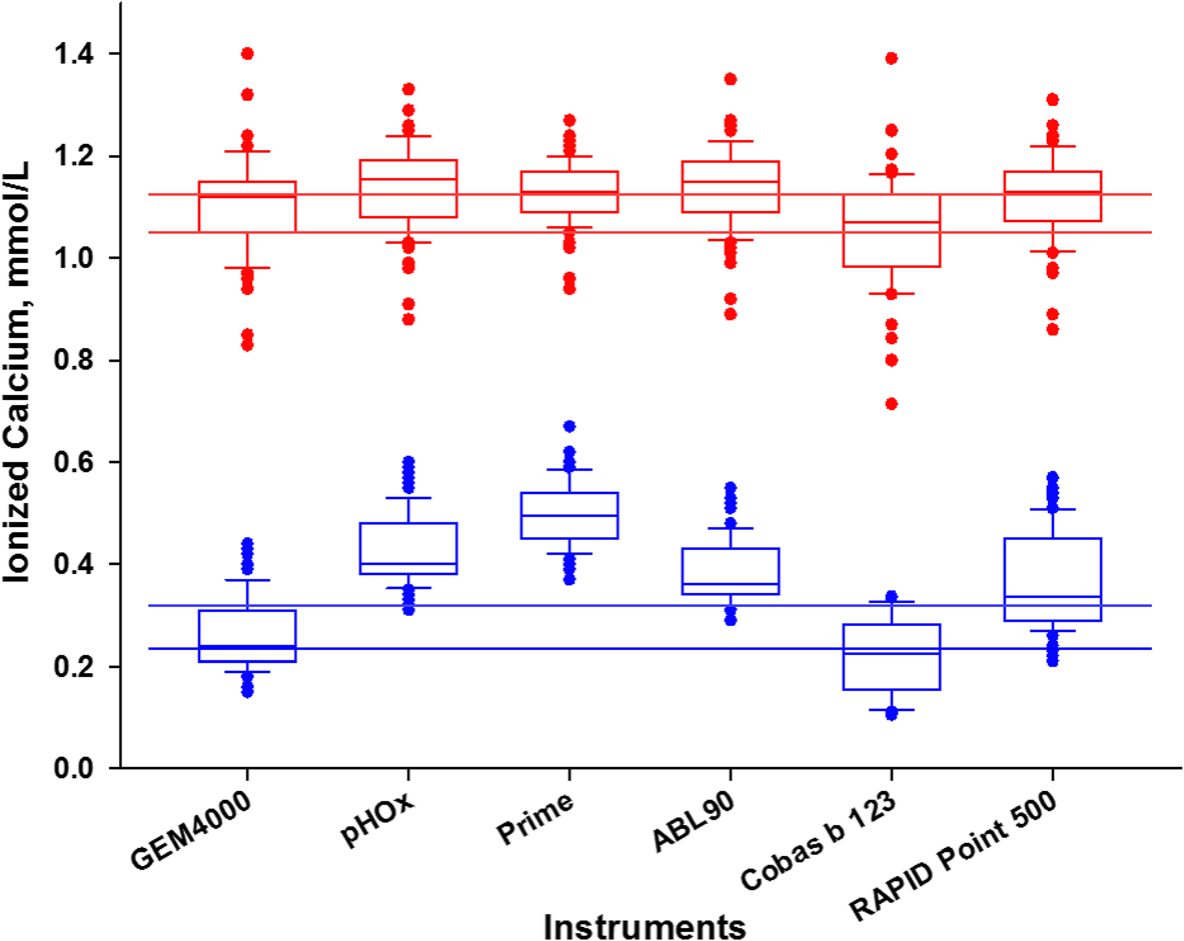
Measurements of ionized calcium illustrated as box plots for systemic and post filter samples by instrument. *Lower row* (*blue boxplots*), post filter samples; *upper row* (*red boxplots*), systemic samples. *Horizontal lines*, respective upper and lower limits of target intervals according to previous publications [7, 8]

Based on the samples that gave results in at least five devices the systemic iCa median concentration ranged from 1.07 to 1.16 mmol/L (Fig. 2) with the median within in the recommended target interval (1.12–1.20 mmol/L) for five of the six devices. Post filter iCa median concentrations of the different devices ranged from 0.21 to 0.50 mmol/L (Fig. 2). There was only one of the six devices with a median value within the target interval of the recommendation scheme (0.24–0.35 mmol/L) [7, 8].

**Fig. 3:**
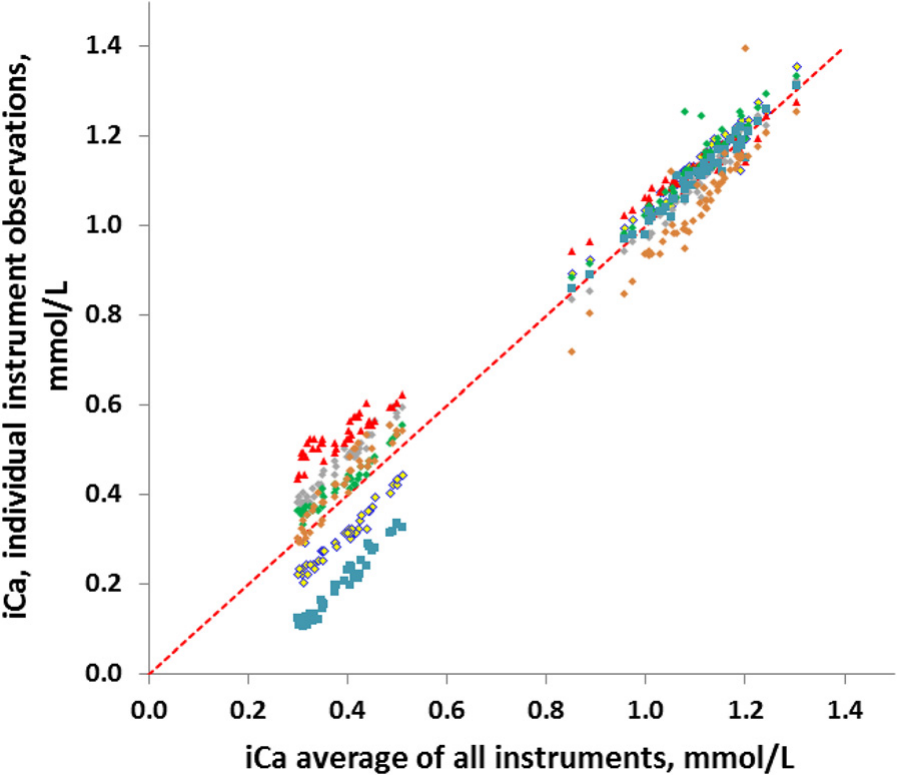
Systemic and post filter measurements of ionized calcium (*iCa*) concentrations. Regressions are between results from individual instruments and the average of all results of a particular sample. The maximum difference in the post filter group was found between the Prime (*red triangles*) and the Cobas b 123 (*blue squares*). The other instruments are (top to bottom, *diamonds*) the pHOx (*gray*), ABL90 (*green*), Rapid (*light brown*) and Gem4000 (*yellow and black border*). *Red dotted line* is the equal line. The relative maximum difference in the systematic result group was miniscule and the individual observations indistinguishable at the present resolution

Differences (Δ) between instruments were calculated as the averages of the individual samples. The maximum difference between two instruments was 0.09 mmol/L (95 % CI 0.02 mmol/L) for systemic samples and 0.33 mmol/L (95 % CI ± 0.01 mmol/L; median Δ 0.29 mmol/L; 0.21–0.50 mmol/L) for post filter samples. In Fig. 3 the observations of the individual instruments are plotted against the average results over all instruments of the same sample for both systemic and poster filter samples.

The clinical impact of the results of all 218 samples was evaluated according to the manufacturers’ recommendations [7, 8]. Results of iCa in the systemic samples are used for regulating calcium flow. In five out of the six devices more than 80 % of the systemic iCa concentrations were within the recommended target interval (1.12–1.20 mmol/L) and the next categories below (1.05–1.11 mmol/L) and above (1.21–1.30 mmol/L; Tables 1 and 2), respectively. For the sixth instrument 56 % of the systemic results were within these three central intervals of the recommendation scheme.

**Table 1:** Recommendations for change of calcium flow based on systemic ionized calcium (iCa) concentration in Ci-Ca® CVVHD and percentage of study samples in each category depending on the instrument used for iCa measurement

Systemic iCa (mmol/L)	Change in calcium dose (calcium/filtrate)	GEM4000 (%)	pHOx (%)	Prime (%)	ABL90 (%)	Cobas b 123 (%)	RAPID point 500 (%)
>1.45	Decreased by 0.6 mmol/L^a^	0	0	0	0	0	0
1.31–1.45	Decreased by 0.4 mmol/L	2	1	0	1	2	2
1.21–1.30	Decreased by 0.2 mmol/L	12	21	11	21	19	3
1.12–1.20	No change	38	41	49	41	51	22
1.05–1.11	Increased by 0.2 mmol/L	27	21	31	21	17	31
0.95–1.11	Increased by 0.4 mmol/L	16	11	6	11	10	25
<0.95	Increased by 0.6 mmol/L^a^	6	4	3	4	2	17

^a^and inform physician

**Table 2:** Recommendations for change of citrate flow based on post filter ionized calcium (iCa) concentration in Ci-Ca® CVVHD and percentage of study samples in each category depending on the instrument used for iCa measurement

Post filter iCa (mmol/L)	Change in citrate dose (citrate/blood)	GEM4000 (%)	pHOx (%)	Prime (%)	ABL90 (%)	Cobas b 123 (%)	RAPID point 500 (%)
>0.45	Increased by 0.3 mmol/L^a^	0	28	72	12	0	22
0.40–0.45	Increased by 0.2 mmol/L	6	24	26	21	0	11
0.35–0.45	Increased by 0.1 mmol/L	6	42	3	36	0	11
0.24–0.35	No change	30	6	0	31	23	49
0.20–0.24	Decreased by 0.1 mmol/L	42	0	0	0	28	6
0.15–0.19	Decreased by 0.2 mmol/L	16	0	0	0	17	0
<0.15	Decreased by 0.3 mmol/L^a^	0	0	0	0	32	0

^a^and inform physician

Results of iCa in post filter samples are used to adjust citrate flow. The percentage of post filter samples within the recommended target interval (0.25–0.34 mmol/L) and the next categories below (0.20–0.24 mmol/L) and above (0.35–0.45 mmol/L) ranged from 3 to 87 % (Tables 1 and 2). However, the distributions were not focused on the recommended target interval but were asymmetric with maximum percentages in almost any category of the scheme depending on the instrument used. If transformed into clinical action, the iCa measurement of the same post filter sample could lead to maximal increase or maximum decrease of the citrate flow depending on the instrument used.

## Discussion

RCA for CRRT represents an established procedure in critically ill patients with contraindications for in vivo anticoagulation [1]. In view of patient safety and performance of CRRT the manufacturer Fresenius Medical Care recommends monitoring iCa concentrations in systemic and post filter samples to adjust calcium and citrate flow. However, the recommendations do not include information on the methods or devices to be used for measuring iCa concentrations. Instruments such as blood gas analyzers are frequently used in monitoring RCA due to their availability. While the iCa concentrations in the systemic samples were within or close to the physiological interval (1.12–1.32 mmol/L) the post filter samples were characterized by iCa concentrations clearly below 0.5 mmol/L [11]. We evaluated iCa measurements of 102 systemic and 116 post filter samples from patients undergoing RCA for CRRT (mulitFiltrate Ci-Ca® Continuous Veno-Venous Hemo Dialysis, CVVHD, Fresenius Medical Care, Bad Homburg, Germany) using six different commercially available blood gas analyzers.

To compensate the continuous loss of calcium and to avoid hypocalcemia during RCA in CRRT the calcium flow starts at 1.7 mmol calcium per liter of effluent. This calcium flow is then increased, left unchanged or decreased according to the systemic iCa results. The systemic iCa concentrations showed a good concordance between the different instruments indicating that they are suitable for controlling iCa substitution, indicating that they are suitable for controlling iCa substitution.

The manufacturer Fresenius Medical Care recommends to either raise, leave unchanged or lower the citrate flow depending on the measured concentration of iCa in post filter samples [7]. Our results indicate that the concentration of iCa measured in post filter samples differ considerably among the instruments as illustrated in Figs. 2 and 3. Presently available technology does not allow defining a reference method or value under post filter measuring conditions. When this ambiguity of the results of measurements is translated into clinical decisions based on the recommendations of Fresenius Medical Care the results from the same post filter sample could vary from a maximum increase of citrate flow (by 0.3 mmol/L) to a maximum decrease of citrate flow (by 0.3 mmol/L) depending on the instrument used. This is also underlined by the differing medians (Fig. 2).

For low iCa in post filter samples it is important to note that we do not know which result represents the best estimate because there is no suitable reference method or commutable reference material available. The detailed scheme given by the manufacturer needs to be adjusted according to the instrument used, and made available to all customers. It is noteworthy that the recommendations are based on a report referring to results from one single patient [7, 8] using an “ABL750” (Radiometer, Copenhagen, Denmark). Furthermore, studies refer to and confirm a target interval for post filter iCa of 0.25–0.35 mmol/L without disclosing the method used for the respective iCa measurement [12, 13].

We therefore propose not to use post filter iCa for controlling RCA in CRRT until a reference method has been established, or at least a thorough evaluation of the different measuring systems in combination with different dialysis systems has been conducted. The maximum average difference (0.33 mmol/L; 95 % CI ± 0.01 mmol/L) or the median difference (Δ 0.29 mmol/L; 0.21–0.50 mmol/L) in post filter iCa concentrations among the investigated devices are approximately as large as the complete interval of the recommendation scheme (Δ 0.30 mmol/L; 0.15–0.45 mmol/L) given by Fresenius Medical Care (Tables 1 and 2) [7, 8]. The investigated instruments are optimized for physiological iCa concentrations, which might make them less suitable for measuring the low iCa in blood with a high concentration of citrate. It has to be noted that post filter samples differ from whole blood in many respects: the material is characterized by low iCa concentrations and it contains citrate, which alters the pH and consequently affects the binding capacity of proteins for calcium. Furthermore, citrate might harm the sensors in the instruments and could affect measurements other than the iCa. As a consequence this could also harm patients not undergoing citrate dialysis and therefore should be subject to further studies.

From a clinical point of view RCA in CRRT can be controlled by using the systemic iCa and disregarding the post filter iCa. The post filter iCa should only be used to prove that the blood is anticoagulated but not to control citrate dose. Lowering citrate flow because of false low post filter iCa concentrations would increase the risk of coagulation in the extracorporeal circuit. Increasing citrate flow because of false high post iCa concentrations might provoke citrate intoxication. Both are potentially life-threatening complications in critically ill patients.

## Conclusion

The recommendations of Fresenius Medical Care need to be revised and adapted to specific instruments for iCa concentration measurements to ensure patient safety. Measurements of iCa in post filter samples should only be used to prove that the blood in the extracorporeal circuit is anticoagulated but not to control citrate dose. Reference methods for low iCa in whole blood containing citrate need to be established.

## Key messages

- The recommendations of Fresenius Medical Care need to be revised and adapted to specific instruments
- Measurements of iCa in post filter samples should only be used to prove that the blood in the extracorporeal circuit is anticoagulated but not to control citrate dose
- Reference methods for low iCa in whole blood containing citrate should be established

## Abbreviations

CRRT: continuous renal replacement therapy
iCa: ionized calcium
IQC: internal quality control
POCT: point of care testing
RCA: regional citrate anticoagulation
Rili-BAEK: Guideline of the German Medical Association on Quality Assurance in Medical Laboratory Examinations
